# Impact of a multimodal hand hygiene intervention on methicillin-resistant *Staphylococcus aureus* carriage among healthcare workers in Libya: a quasi-experimental pre–post study

**DOI:** 10.1186/s13756-026-01721-y

**Published:** 2026-02-17

**Authors:** Mohanned Mohamed Alwashaish

**Affiliations:** Microbiology Department, Faculty of Pharmacy, Misurata University, Misurata, Libya

**Keywords:** Hand hygiene, MRSA, Healthcare workers, Infection prevention and control, Quasi-experimental, MAR index, Multidrug resistance, LMIC, Libya

## Abstract

**Background:**

Healthcare-associated methicillin-resistant Staphylococcus aureus (MRSA) remains a major threat in low-resource settings, where interventional evidence linking improvements in hand hygiene (HH) to changes in MRSA carriage among healthcare workers (HCWs) is limited. We evaluated the association between implementation of a multimodal HH program and MRSA carriage in a tertiary-care hospital in Libya.

**Methods:**

We conducted a quasi-experimental pre–post study (January–December 2024) across four hospital units (intensive care unit, neonatal intensive care unit, surgical ward, and radiology department). Paired anterior nares swabs were collected from HCWs at baseline and one year after intervention implementation. MRSA was identified using cefoxitin disk diffusion according to CLSI 2024 guidelines. Antimicrobial susceptibility testing was performed by the Kirby–Bauer method, and vancomycin minimum inhibitory concentrations were determined using E-test. HH compliance was assessed using WHO observational methods. The primary analysis used McNemar’s test and generalized estimating equation (GEE) logistic regression, while multidrug resistance was summarized using the multiple antibiotic resistance (MAR) index.

**Results:**

Among 318 paired HCWs, MRSA carriage decreased from 31.1 (99/318) at baseline to 19.5% (62/318) post-intervention (absolute reduction of 11.6 percentage points; *p* < 0.001). The adjusted odds of MRSA carriage post-intervention were 0.52 (95% CI 0.36–0.75; *p* < 0.001). HH compliance improved from 42.7% (486/1139 observed opportunities) to 71.3% (801/1123; *p* < 0.001). Although individual antimicrobial resistance proportions remained largely unchanged, the median MAR index decreased from 0.42 (IQR 0.33–0.50) to 0.33 (IQR 0.25–0.42) (*p* < 0.001), indicating a reduction in the overall multidrug resistance burden among circulating MRSA isolates. No vancomycin resistance was detected.

**Conclusions:**

Implementation of a multimodal WHO-aligned HH intervention was associated with a significant reduction in MRSA carriage among HCWs and a lower overall multidrug resistance burden, as reflected by the MAR index. These findings provide pragmatic, low-cost evidence to support strengthening infection prevention and control programs in Libya and comparable low- and middle-income country settings.

**Supplementary Information:**

The online version contains supplementary material available at 10.1186/s13756-026-01721-y.

## Introduction

### Background and rationale

Healthcare-associated infections (HAIs) are a global health concern, causing significant morbidity, mortality, and economic losses. In the European Union/European Economic Area alone, antibiotic-resistant bacteria accounted for more than 800,000 disability-adjusted life-years (DALYs) between 2015 and 2019 [1]. Among the major pathogens, methicillin-resistant *Staphylococcus aureus* (MRSA) is recognized by the WHO as a critical priority organism due to its high burden of bloodstream infections, pneumonia, and surgical site infections [2].

Healthcare workers (HCWs) play a pivotal role in the epidemiology of MRSA, serving both as reservoirs and vectors of transmission. Nasal and hand carriage among HCWs has been repeatedly identified as a risk factor for cross-transmission to patients, especially in intensive care and surgical settings [3–5]. Reported MRSA carriage rates vary considerably, ranging from 2 to 15% in high-income countries to > 30% in low- and middle-income countries (LMICs) [6]. Recent systematic reviews indicate that in Africa, MRSA carriage among HCWs averages ~ 21% [7], while studies in Libya and Tunisia report rates as high as 25–35% [8, 9].

Hand hygiene (HH) is universally recognized as the cornerstone of infection prevention and control (IPC). The WHO Multimodal Hand Hygiene Strategy—comprising system change, training, reminders, direct observation/feedback, and institutional climate has been shown to improve HH compliance and reduce HAIs in both high- and low-resource settings [10, 11]. However, most existing research has focused on self-reported practices or compliance audits, with few studies directly linking HH interventions to reductions in MRSA carriage among HCWs in LMICs [12].

### Knowledge gap

Despite the high burden of antimicrobial resistance in North Africa, no interventional study has assessed whether a multimodal HH program can reduce MRSA carriage among HCWs in Libyan hospitals. Existing data are largely cross-sectional and limited to prevalence surveys [8]. This restricts evidence-based guidance for IPC programs in resource-constrained healthcare systems.

To our knowledge, this is the first study from Libya and one of very few from North Africa designed to evaluate the impact of a multimodal hand hygiene strategy on MRSA carriage among healthcare workers.

### Objectives

The objectives of this study were to determine the prevalence of MRSA carriage among healthcare workers before and after a multimodal hand-hygiene intervention, to evaluate changes in hand-hygiene compliance using WHO observational methods, and to characterize antimicrobial resistance patterns of MRSA isolates, including calculation of the multiple antibiotic resistance index. By addressing these objectives, this study seeks to provide the first interventional evidence from Libya linking HH improvement to MRSA carriage reduction among HCWs, thereby contributing to both national IPC strategies and the global AMR agenda.

## Materials and methods

### Study design

This was a quasi-experimental pre–post intervention study designed to evaluate the effect of a multimodal hand-hygiene intervention on MRSA carriage among healthcare workers (HCWs). The study followed the STROBE recommendations for reporting observational studies.

### Setting

The study was conducted at Misurata Central Hospital, Libya, across four clinical units: the Intensive Care Unit (ICU), Surgical Ward, Neonatal Intensive Care Unit (NICU), and Radiology Department. The study period was January–December 2024.

### Participants

#### Eligibility criteria

Healthcare workers were eligible for inclusion if they were employed in one of the participating wards during the study period, were actively involved in direct patient care (including physicians, nurses, and allied health professionals/technicians), had been working in the ward for at least one month prior to enrolment, and provided verbal informed consent. Healthcare workers were excluded if they had received systemic antibiotics, topical intranasal antibiotics, or MRSA decolonization therapy (e.g., mupirocin) within two weeks prior to sampling, had extended leave or no direct patient contact during the study period, or declined to participate.

#### Number of participants

A total of 318 HCWs were enrolled and sampled at both baseline and post-intervention time points.

#### Variables

The primary outcome of the study was MRSA carriage, defined as the presence of MRSA in anterior nasal swabs. Secondary outcomes included hand-hygiene compliance measured using WHO observational methods, antimicrobial resistance profiles of MRSA isolates, and the multiple antibiotic resistance (MAR) index calculated for each isolate. Covariates considered in the analysis included age, sex, years of professional experience, profession (nurse, physician, or technician), ward, and work shift.

### Data sources and measurement

#### Sampling and microbiological procedures

Anterior nasal swabs were collected from all participants at both baseline and post-intervention time points and constituted the primary measure of MRSA carriage. Hand swabs were collected from a predefined subset of healthcare workers to assess transient hand contamination. Hand-swab results were analyzed descriptively and were not used to define persistent MRSA carriage.

Swabs were inoculated onto Mannitol Salt Agar (Oxoid, UK) and incubated at 35 ± 2 °C for 24–48 h. *S. aureus* colonies were confirmed by Gram stain, catalase, and tube coagulase tests, supplemented by API Staph (bioMérieux, France) where necessary [13, 14].

MRSA identification was performed using cefoxitin (30 µg) disk diffusion per CLSI M100, 34th edition, 2024 [15].

Antimicrobial susceptibility testing (AST) was conducted by Kirby–Bauer disk diffusion on Mueller–Hinton agar following CLSI guidelines [15]. Antibiotics tested: penicillin, cefoxitin, erythromycin, clindamycin (with D-test), tetracycline, ciprofloxacin, gentamicin, trimethoprim–sulfamethoxazole, and vancomycin MIC determined by E-test (bioMérieux, France) [15, 16].

Quality control (QC) strains: *S. aureus* ATCC 25923 and *E. coli* ATCC 25922 were included in each AST batch [15].

MDR definition: resistance to ≥ 1 agent in ≥ 3 antimicrobial categories [17].

MAR index: number of antibiotics resisted / total tested [18].

### Hand-hygiene intervention and measurement

The WHO Multimodal Hand Hygiene Strategy was implemented, including system change, education and training, workplace reminders, monitoring with feedback, and promotion of an institutional safety climate [19]. System change involved ensuring the continuous availability of alcohol-based hand rub at points of care. Education and training consisted of structured sessions delivered to healthcare workers on the WHO “My 5 Moments for Hand Hygiene” [20]. Workplace reminders included posters and visual cues displayed in clinical areas to reinforce correct hand-hygiene practices. Monitoring and feedback were conducted through direct observation by trained observers, with periodic feedback provided to staff. Promotion of an institutional safety climate was supported through leadership engagement and regular staff meetings emphasizing the importance of hand hygiene as a core component of infection prevention and control.

Hand-hygiene compliance was assessed using the WHO Observation Form. Trained observers recorded hand-hygiene opportunities and actions, and compliance was calculated as the proportion of performed actions divided by the total number of observed opportunities, expressed as a percentage. The WHO Hand Hygiene Self-Assessment Framework (HHSAF) was not formally applied; progress was evaluated using repeated direct observation of hand hygiene compliance.

The “before” period corresponded to baseline observations conducted prior to implementation of the intervention, whereas the “after” period reflected observations conducted one year after full implementation.

### Efforts to address potential sources of bias

Several measures were implemented to minimize potential sources of bias. Sampling and laboratory procedures were standardized and applied consistently during both baseline and post-intervention periods. Laboratory personnel were blinded to the timing of isolate collection to reduce measurement bias. Hand-hygiene observations included unannounced sessions to mitigate the Hawthorne effect. Quality-control strains were used routinely to ensure consistency and reliability of antimicrobial susceptibility testing. In addition, no other structured infection prevention or control interventions, such as active MRSA screening, decolonization protocols, antimicrobial stewardship programs, or enhanced environmental cleaning initiatives, were introduced or modified during the study period. Routine IPC practices remained unchanged, and the multimodal hand-hygiene program represented the only structured intervention implemented hospital-wide.

### Study size

The study included 318 paired participants. Based on a baseline MRSA prevalence of 31%, this sample size provided > 80% power (α = 0.05) to detect an absolute reduction of ~ 8–10 percentage points in MRSA carriage between pre- and post-intervention.

### Quantitative variables

MRSA carriage was treated as a binary outcome variable (present or absent). Hand-hygiene compliance was expressed as a percentage calculated from the number of performed actions divided by the total number of observed opportunities. The MAR index was analyzed as a continuous variable ranging from 0 to 1 and summarized using median and interquartile range. Demographic variables included age and years of professional experience as continuous variables, while sex, profession, ward, and work shift were analyzed as categorical variables.

### Statistical methods

The primary outcome, MRSA carriage before and after the intervention, was analyzed using McNemar’s test for paired proportions. In addition, generalized estimating equation (GEE) logistic regression models with a logit link and exchangeable correlation structure were used to estimate adjusted odds ratios (aORs) with 95% confidence intervals for the intervention effect. Models were adjusted for potential confounders, including sex, age, years of professional experience, profession, and ward.

Secondary outcomes were analyzed as follows. Hand-hygiene compliance was modeled using binomial GEE, with the number of performed actions as the numerator and the number of observed opportunities as the denominator, and results were expressed as percentage-point changes. Changes in the Multiple Antibiotic Resistance (MAR) index were assessed using the Wilcoxon signed-rank test, with bootstrap-derived 95% confidence intervals for the change in median values. Co-resistance patterns were explored using correlation matrices and hierarchical clustering based on Ward’s method and Jaccard distance.

Antibiotic-specific resistance proportions were compared between baseline and post-intervention isolates using χ^2^ or Fisher’s exact tests, as appropriate. Healthcare workers with missing follow-up swabs (n = 8) were excluded from the paired primary analysis but were included in sensitivity analyses. Sensitivity analyses also included per-protocol analyses and exclusion of healthcare workers who changed ward or role during the study period; multiple imputation using chained equations (MICE) was planned if missing data exceeded 5%.

To account for multiple comparisons in secondary outcomes, *p*-values were adjusted using the Benjamini–Hochberg false discovery rate procedure. All analyses were performed using SPSS version 26.0 and R version 4.3.2, with a two-sided significance level of α = 0.05.

### Ethical considerations

The study was approved by the faculty of pharmacy (Approval No: 13/2024). verbal informed consent was obtained from all participants. Confidentiality and anonymity were preserved.

## Results

### Participants

A total of 318 healthcare workers (HCWs) were enrolled across four hospital units: ICU (n = 102), Surgical Ward (n = 88), NICU (n = 74), and Radiology Department (n = 54).

### Descriptive data

The median age was 34 years (IQR 28–42), with 58% females and 42% males. Nurses constituted 61% of participants, physicians 23%, and technicians 16%. The median duration of professional experience was 8 years (IQR 4–14) as presented in Table 1.

**Table 1:** Baseline characteristics of healthcare workers

Characteristic	ICU (n = 102)	Surgical (n = 88)	NICU (n = 74)	Radiology (n = 54)	Total (N = 318)
Median age, years (IQR)	35 (29–43)	33 (28–41)	32 (27–40)	36 (30–45)	34 (28–42)
Female sex, n (%)	58 (56.9)	53 (60.2)	49 (66.2)	25 (46.3)	185 (58.2)
Profession: nurse, n (%)	68 (66.7)	51 (58.0)	44 (59.5)	30 (55.6)	193 (60.7)
Profession: physician, n (%)	20 (19.6)	21 (23.9)	18 (24.3)	14 (25.9)	73 (22.9)
Profession: technician, n (%)	14 (13.7)	16 (18.2)	12 (16.2)	10 (18.5)	52 (16.4)
Median years of experience (IQR)	9 (5–15)	7 (4–12)	6 (3–11)	10 (6–15)	8 (4–14)

No significant differences in age, sex, or profession across wards (Kruskal–Wallis *p* = 0.12; χ^2^ *p* = 0.18).

### Outcome data

#### MRSA carriage

All MRSA carriage estimates reported in this study refer exclusively to nasal carriage. At baseline, 99 of 318 healthcare workers (31.1%) were colonized with MRSA, with the highest prevalence observed in the ICU (37.3%) and NICU (32.4%), followed by the Surgical Ward (27.3%) and the Radiology Department (24.1%). After one year of implementing the multimodal hand-hygiene intervention, MRSA carriage significantly decreased to 62 of 318 healthcare workers (19.5%), as shown in Table 2 (absolute reduction of 11.6 percentage points; *p* < 0.001 by McNemar’s test). Among paired healthcare workers, 54 remained MRSA-positive at both time points, 45 converted from MRSA-positive to MRSA-negative, 8 converted from MRSA-negative to MRSA-positive, and 211 remained MRSA-negative throughout the study period.

**Table 2:** MRSA carriage among healthcare workers before and after hand-hygiene intervention

Ward	Baseline MRSA n (%)	Post-intervention MRSA n (%)	Absolute change (%)	*p*-value (McNemar)
ICU (n = 102)	38 (37.3)	22 (21.6)	− 15.7	0.004
Surgical (n = 88)	24 (27.3)	15 (17.0)	− 10.3	0.041
NICU (n = 74)	24 (32.4)	17 (23.0)	− 9.4	0.072
Radiology (n = 54)	13 (24.1)	8 (14.8)	− 9.3	0.128
Total (N = 318)	99 (31.1)	62 (19.5)	− 11.6	< 0.001

McNemar’s test applied for paired analysis; Bonferroni-adjusted α = 0.0125 for ward-level comparisons.

### Main results

#### Hand-hygiene compliance

Overall hand-hygiene compliance improved from 42.7% (486/1139 observed opportunities) at baseline to 71.3% (801/1123) post-intervention, corresponding to an absolute increase of 28.6 percentage points (*p* < 0.001 by binomial GEE). The most marked improvements were observed for the WHO moments “after patient contact” (from 39 to 74%) and “before aseptic procedure” (from 45 to 72%).

Improvements in hand-hygiene compliance were observed across all wards (Table S1). The largest increase was recorded in the intensive care unit (ICU), where compliance rose from 38.5% (154/400) to 74.8% (305/408; Δpp = + 36.3). Compliance also increased in the Surgical Ward from 44.2% (138/312) to 71.5% (221/309; Δpp = + 27.3), in the neonatal intensive care unit (NICU) from 43.1% (124/288) to 69.6% (199/286; Δpp = + 26.5), and in the Radiology Department from 47.6% (70/147) to 63.6% (76/120; Δpp = + 16.0).

When stratified by professional category (Table S2), hand-hygiene compliance improved among all groups. Compliance among nurses increased from 45.1% (302/670) at baseline to 75.9% (511/673) post-intervention (Δpp = + 30.8). Among physicians, compliance rose from 40.3% (118/293) to 68.4% (199/291; Δpp = + 28.1), while technicians showed an increase from 36.1% (66/183) to 61.4% (91/148; Δpp = + 25.3).

Across professional categories, nurses demonstrated the largest absolute improvement in hand-hygiene compliance, followed by physicians and technicians, reflecting differences in baseline compliance levels and patterns of patient contact.

### Sensitivity analysis

Results remained robust in sensitivity analyses excluding HCWs who transferred wards (n = 12) or had missing follow-up swabs (n = 8). Adjusted GEE models confirmed the intervention effect (aOR for MRSA carriage post-intervention = 0.52; 95% CI 0.36–0.75; *p* < 0.001).

### Antimicrobial resistance profiles

**Fig. 1:**
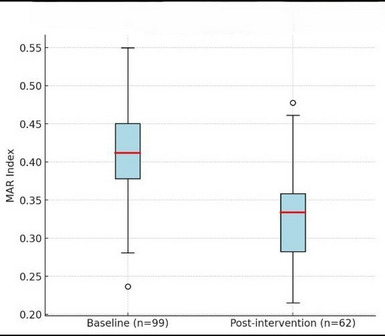
Distribution of Multiple Antibiotic Resistance (MAR) index among MRSA isolates before and after hand-hygiene intervention

Among baseline MRSA isolates (n = 99), high resistance was observed to penicillin (100%), erythromycin (58%), and ciprofloxacin (42%). Post-intervention isolates (n = 62) showed similar resistance trends, but the MAR index decreased significantly from median 0.42 (IQR 0.33–0.50) to 0.33 (0.25–0.42) (*p* < 0.001 by Wilcoxon signed-rank test) as shown in Table 3; Fig. 1.

**Table 3:** Antimicrobial resistance patterns of MRSA isolates before and after intervention

Antibiotic	Baseline (n = 99) n (%)	Post-intervention (n = 62) n (%)	*p*-value (χ^2^ or Fisher)
Penicillin	99 (100)	62 (100)	–
Cefoxitin (MRSA confirmatory)	99 (100)	62 (100)	–
Erythromycin	57 (57.6)	31 (50.0)	0.34
Clindamycin (D-test pos.)	38 (38.4)	19 (30.6)	0.28
Tetracycline	24 (24.2)	13 (21.0)	0.66
Ciprofloxacin	42 (42.4)	20 (32.3)	0.19
Gentamicin	28 (28.3)	16 (25.8)	0.73
TMP-SMX	19 (19.2)	12 (19.4)	0.97
Vancomycin (MIC > 2 µg/mL)	0 (0)	0 (0)	–

*TMP-SMX* trimethoprim–sulfamethoxazole, *MIC* Minimum inhibition concentration

Correlation analysis based on Ward’s hierarchical clustering using Jaccard distance revealed strong co-resistance between erythromycin and clindamycin, and between ciprofloxacin and gentamicin.

Boxplots represent median and IQR of MAR index values across isolates at baseline (n = 99) and post-intervention (n = 62). Whiskers denote 1.5 × IQR. A significant reduction was observed (*p* < 0.001, Wilcoxon signed-rank test).

## Discussion

This study demonstrated that implementation of a multimodal hand-hygiene (HH) intervention was followed by a substantial reduction in MRSA carriage among healthcare workers (HCWs) in a tertiary Libyan hospital. Importantly, while individual resistance profiles remained stable, the overall burden of multidrug resistance, as reflected in the MAR index, decreased significantly. These findings underscore the importance of IPC programs as an essential pillar of antimicrobial resistance (AMR) containment in resource-limited settings.

### Prevalence of MRSA carriage among HCWs

The high baseline MRSA carriage observed in this study reflects the persistent challenges facing IPC programs in North Africa. Several factors may explain this elevated prevalence: limited resources for screening, overcrowded wards, inadequate nurse-to-patient ratios, and frequent empirical use of broad-spectrum antibiotics without stewardship oversight.

Comparable prevalence has been reported in Libya, where Elramalli et al. found nasal MRSA carriage among more than one-quarter of HCWs [21]. In Tunisia, Ben Sallem et al. reported similarly high rates, particularly among surgical staff [22]. A systematic review across Africa confirmed that MRSA carriage among HCWs averages ~ 21%, but hospitals in North Africa frequently exceed this figure [23]. In Egypt, El-Kholy et al. documented MRSA prevalence approaching 30% [24], while in Saudi Arabia, Balkhy et al. observed lower rates of 12–18% [25]. Sub-Saharan African studies report even higher prevalence, including Ethiopia (32–36%) [26] and Nigeria (> 35%) [27].

By contrast, carriage in Europe rarely exceeds 5–10% [28], reflecting stricter antimicrobial stewardship, systematic MRSA screening, and mature IPC programs. Thus, the high prevalence in our setting is consistent with other LMICs and underscores the urgent need for context-adapted IPC interventions. This high prevalence has critical implications for patient safety, particularly in ICUs and NICUs, where HCW carriage is a well-established driver of outbreaks, excess morbidity, and healthcare costs. Importantly, no decolonization procedures were performed for MRSA-colonized healthcare workers during the study period; therefore, the observed reduction in MRSA carriage following the intervention likely reflects decreased acquisition and transmission rather than eradication of long-standing colonization.

### Impact of multimodal HH intervention

The intervention produced a significant reduction in MRSA carriage across wards, particularly in high-risk units such as ICUs. This effect can be explained by the multimodal nature of the strategy, which combined infrastructure changes, structured training, reminders, feedback, and fostering of safety culture. Each component reinforced the others, producing a sustainable improvement in HH behavior.

Our findings align with global literature. Allegranzi et al. demonstrated that the WHO multimodal HH strategy improved compliance and reduced HAIs in a multicenter quasi-experimental trial [29]. In Middle Eastern hospitals, Mahfouz et al. reported substantial gains in compliance following structured training [30]. Similarly, interventions in India [31] and Pakistan [32] confirmed that multimodal HH promotion can reduce MRSA acquisition and transmission.

The biological rationale is clear: MRSA spreads primarily through hand contact, and improved HH disrupts this pathway [33]. Importantly, while resistance phenotypes remained stable, the observed reduction in the MAR index indicates a decrease in the overall multidrug resistance burden among circulating MRSA isolates, rather than evidence of specific clonal replacement., as evidenced by the drop in MAR index. This distinction indicates that IPC primarily affects transmission dynamics, whereas antimicrobial stewardship is required to modify selection pressures.

Alternative explanations should be considered. The Hawthorne effect may partly explain improvements in observed hand-hygiene compliance, as healthcare workers were aware of observation. However, this effect primarily influences behavior and does not directly explain changes in MRSA nasal carriage.

Although molecular typing was not performed, the consistent reduction in both MRSA carriage and MAR index across wards suggests a reduction in the overall multidrug resistance burden among circulating MRSA isolates, supporting the epidemiological impact of the intervention.

### Antimicrobial resistance profiles

Our study found universal resistance to β-lactams and high resistance to macrolides and fluoroquinolones, which is consistent with previous Libyan and Tunisian reports [21, 22]. In Algeria, Bouchami et al. also identified high resistance to erythromycin and ciprofloxacin in MRSA isolates, though vancomycin susceptibility was retained [34]. Egyptian data confirm similar trends, with Abouelfetouh et al. reporting high macrolide resistance but preserved vancomycin sensitivity [35].

Globally, resistance levels vary widely. European surveillance data show ~ 35% erythromycin resistance [36], whereas African hospitals frequently exceed 50% [23]. These differences reflect prescribing patterns: fluoroquinolone and macrolide overuse is common in North Africa, selecting for resistant strains.

The absence of vancomycin resistance is reassuring and aligns with Tunisian and Egyptian findings [22, 35]. Nonetheless, reduced susceptibility (VISA) and VRSA strains have been reported in Asia and North America [37], underscoring the importance of ongoing monitoring.

The significant decline in MAR index is particularly noteworthy. While individual antibiotic resistance proportions did not change, the MAR index dropped, indicating fewer multidrug-resistant, which are often highly resistant. MAR index therefore offers a more sensitive epidemiological signal than binary resistance outcomes [18].

This study is also among the few from LMICs to longitudinally apply the MAR index, highlighting its potential as a practical surveillance tool to complement traditional resistance reporting in resource-limited settings.

### Clinical and public health implications

These findings highlight the potential of hand hygiene as a low-cost and scalable intervention in resource-limited healthcare settings. Improved hand-hygiene compliance may have important implications for patient safety by reducing opportunities for MRSA transmission. In addition, the results are relevant for policymakers seeking pragmatic IPC strategies aligned with national and global antimicrobial resistance action plans.

## Strengths, limitations, and future directions

This study has several notable strengths. It represents the first interventional evidence from Libya linking multimodal hand-hygiene promotion to reductions in MRSA carriage among healthcare workers. The large sample size, prospective quasi-experimental design, use of standardized CLSI 2024 microbiological methods with quality-control strains, and advanced statistical modeling (including MAR index and GEE analysis) all strengthen the reliability of the findings.

Nonetheless, several limitations should be acknowledged. First, the quasi-experimental before–after design inherently limits causal inference. Although no additional structured infection prevention or control (IPC) interventions, such as active MRSA screening, decolonization protocols, antimicrobial stewardship programs, or enhanced environmental cleaning initiatives, were introduced or modified during the study period, unmeasured secular trends or behavioral changes cannot be fully excluded. Therefore, the observed reduction in MRSA carriage should be interpreted as an association temporally related to the hand-hygiene intervention rather than definitive proof of causality.

Second, the study was conducted in a single tertiary-care hospital and included a limited number of clinical units, which may restrict generalizability to other healthcare settings. However, Misurata Central Hospital is one of the largest referral centers in central Libya, serving a broad and diverse patient population, which supports the external relevance of the findings within similar resource-limited contexts.

Third, routine surveillance data on MRSA carriage or infection among hospitalized patients were not systematically available during the study period. Therefore, patient-level MRSA prevalence could not be assessed, and the present analysis focused exclusively on healthcare worker carriage.

Fourth, MRSA carriage was primarily assessed using nasal swabs, which represent persistent colonization, whereas hand swabs—collected from a subset of participants—reflect transient contamination. Although nasal carriage was used exclusively for the primary outcome, differences between stable colonization and short-term hand contamination may still influence interpretation of transmission dynamics.

Fifth, molecular typing or genomic characterization of MRSA isolates was not performed because of limited laboratory resources and lack of funding, a common constraint in low- and middle-income countries. As a result, inferences regarding clonal relatedness or specific transmission pathways could not be made. Consequently, changes in the MAR index should be interpreted as reflecting shifts in the overall multidrug resistance burden among circulating isolates rather than evidence of specific clonal replacement.

In addition, improvements in observed hand-hygiene compliance may have been partially influenced by the Hawthorne effect. However, this effect primarily impacts observed behavior and does not directly explain changes in MRSA nasal carriage. Post-intervention hand-hygiene observations were not systematically continued beyond the study follow-up period; therefore, the long-term sustainability of the observed compliance improvements could not be formally assessed.

Future research should extend this work to multiple hospitals across Libya and the broader North African region to enhance generalizability. Incorporating molecular typing or genomic surveillance would allow more precise characterization of MRSA transmission dynamics, while implementation science approaches could help evaluate sustainability and contextual factors influencing long-term hand-hygiene adherence. Finally, cost-effectiveness analyses are needed to support policymakers in prioritizing IPC investments in resource-limited healthcare systems.

## Conclusions

This study demonstrates that implementation of a multimodal hand-hygiene intervention was associated with a significant reduction in MRSA carriage among healthcare workers, alongside a decrease in the overall multidrug resistance burden as reflected by the MAR index. These findings underscore the importance of infection prevention and control programs in addressing antimicrobial resistance in resource-limited healthcare settings.

Sustained investment in hand-hygiene promotion, as part of a broader IPC framework, may contribute to reducing MRSA transmission risk in high-burden hospitals. Scaling up similar multimodal interventions could represent a cost-effective component of national antimicrobial resistance action plans in Libya and other low- and middle-income countries, particularly when implemented alongside complementary strategies such as antimicrobial stewardship.

## Supplementary Information

Below is the link to the electronic supplementary material.

Supplementary Material 1.

## Data Availability

The datasets generated and/or analyzed during the current study are available from the corresponding author on reasonable request.
