# Supplementary material for: Impact of a multimodal hand hygiene intervention on methicillin-resistant Staphylococcus aureus carriage among healthcare workers in Libya: a quasi-experimental pre–post study
Source: Antimicrob Resist Infect Control. 2026 Feb 17;15:39. doi: 10.1186/s13756-026-01721-y (PMC13014959; doi:10.1186/s13756-026-01721-y)
Supplement: Supplementary file 1 — Supplementary Material 1. [file 13756_2026_1721_MOESM1_ESM.docx]

Table S1. Hand-hygiene compliance by ward before and after the intervention

| Ward | Baseline compliance % (n/N) | Post-intervention compliance % (n/N) | Δ percentage points |
| --- | --- | --- | --- |
| ICU | 38.5 (154/400) | 74.8 (305/408) | +36.3 |
| Surgical Ward | 44.2 (138/312) | 71.5 (221/309) | +27.3 |
| NICU | 43.1 (124/288) | 69.6 (199/286) | +26.5 |
| Radiology | 47.6 (70/147) | 63.6 (76/120) | +16.0 |
| Total | 42.7 (486/1139) | 71.3 (801/1123) | +28.6 |

Table S2. Hand-hygiene compliance by professional category before and after the intervention

| Profession | Baseline compliance % (n/N) | Post-intervention compliance % (n/N) | Δ percentage points |
| --- | --- | --- | --- |
| Nurses | 45.1 (302/670) | 75.9 (511/673) | +30.8 |
| Physicians | 40.3 (118/293) | 68.4 (199/291) | +28.1 |
| Technicians | 36.1 (66/183) | 61.4 (91/148) | +25.3 |
| Total | 42.7 (486/1139) | 71.3 (801/1123) | +28.6 |
